# AZT resistance alters enzymatic properties and creates an ATP-binding site in SFVmac reverse transcriptase

**DOI:** 10.1186/s12977-015-0147-7

**Published:** 2015-02-22

**Authors:** Anna Schneider, Kristian Schweimer, Paul Rösch, Birgitta M Wöhrl

**Affiliations:** Universität Bayreuth, Lehrstuhl Biopolymere, Universitätsstr. 30, D-95447 Bayreuth, Germany

**Keywords:** AZT resistance, ATP binding, Reverse transcriptase, NMR, Foamy virus, HIV

## Abstract

**Background:**

The replication of simian foamy virus from macaques can be inhibited by the nucleoside reverse transcriptase inhibitor azidothymidine (AZT, zidovudine). Four substitutions in the protease-reverse transcriptase (PR-RT) protein (K211I, I224T, S345T, E350K) are necessary to obtain highly AZT resistant and fully replication competent virus. AZT resistance is based on the excision of the incorporated AZTMP in the presence of ATP. I224T is a polymorphism which is not essential for AZT resistance per se, but is important for regaining efficient replication of the resistant virus.

**Results:**

We constructed PR-RT enzymes harboring one to four amino acid substitutions to analyze them biochemically and to determine their ability to remove the incorporated AZTMP. S345T is the only single substitution variant exhibiting significant AZTMP excision activity. Although K211I alone showed no AZTMP excision activity, excision efficiency doubled when K211I was present in combination with S345T and E350K. K211I also decreased nucleotide binding affinity and increased fidelity. NMR titration experiments revealed that a truncated version of the highly AZT resistant *mt4* variant, comprising only the fingers-palm subdomains was able to bind ATP with a K_*D*_-value of ca. 7.6 mM, whereas no ATP binding could be detected in the corresponding wild type protein. We could show by NMR spectroscopy that S345T is responsible for ATP binding, probably by making a tryptophan residue accessible.

**Conclusion:**

Although AZT resistance in SFVmac is based on excision of the incorporated AZTMP like in HIV-1, the functions of the resistance substitutions in SFVmac PR-RT appear to be different. No mutation resulting in an aromatic residue like F/Y215 in HIV, which is responsible for π-π-stacking interactions with ATP, is present in SFVmac. Instead, S345T is responsible for creating an ATP binding site, probably by making an already existing tryptophan more accessible, which in turn can interact with ATP. This is in contrast to HIV-1 RT, in which an ATP binding site is present in the WT RT but differs from that of the AZT resistant enzyme.

**Electronic supplementary material:**

The online version of this article (doi:10.1186/s12977-015-0147-7) contains supplementary material, which is available to authorized users.

## Background

Simian foamy virus from macaques (SFVmac) belongs to the subfamily of *Spumaretrovirinae,* which, together with the *Orthoretrovirinae* form the family of *Retroviridae* [1]. A considerable difference between the two subfamilies concerns the nature of the cleavage products of the Pol protein, which consists of the protease (PR), reverse transcriptase (RT) and integrase (IN) domains. While in orthoretroviruses the viral enzymes are cleaved from a Gag-Pol precursor protein into PR, RT and IN, foamy viruses (FVs) possess a separate Pol precursor and do not cleave off the PR domain from the RT. Thus, the mature FV enzymes are PR-RT and IN [2,3]. The mature PR-RT is a monomeric protein with the PR (101 amino acid residues) located at its N-terminus [3]. The PR is separated from the putative N-terminus of the RT domain via a stretch of 41 amino acid residues (region 102–143) whose presence appears to be important for RT integrity and proper orientation of the PR domain, which needs to dimerize in order to be active [4-7].

So far, only the human immunodeficiency (HIV) nucleotide RT inhibitor (NtRTI) tenofovir and the nucleoside RT inhibitor (NRTI) azidothymidine (AZT, zidovudine) have been shown to inhibit SFVmac replication [8-11]. Both substances are used for treating patients suffering from HIV infections.

Interestingly, both SFVmac and HIV-1 RT follow a similar AZT resistance mechanism: the incorporated chain terminating AZT-monophosphate (AZTMP) is excised in the presence of ATP [12-14]. It has been shown that the pyrophosphate donor in AZT resistant HIV-1 and SFVmac is ATP [12-14]. In HIV-1, the excision reaction products are a dinucleoside tetraphosphate (AZT-P_4_-A) derived from ATP and AZTMP at the primer terminus and the resulting unblocked 3’OH primer [12]. Formation of the phosphodiester bond between ATP and AZTMP also requires specific ATP binding near the polymerase active site which orients the β,γ-phosphate moiety of ATP proximal to the phosphate group of AZTMP. Removal of AZTMP finally leads to primer rescue and elongation with natural dNTPs [15-17].

In SFVmac PR-RT four amino acid exchanges (K211I, I224T, S345T, E350K) are necessary to confer high resistance against AZT. Amino acid sequence alignments of the polymerase domains of SFVmac and HIV-1 revealed that the SFVmac AZT resistance mutations do not correspond to the ones obtained with highly AZT-resistant HIV-1 RT (M41L, D67N, K70R, T215Y/F and K219Q/E) [12-14,17-21]. In HIV-1 the amino acid exchange T215Y/F is crucial for binding of ATP in the AZT resistant mutant RT. X-ray crystallography data revealed that both the WT and the AZT resistant RT are capable of ATP binding, however at different sites (site I and site II). The aromatic amino acid exchange T215F/Y in the resistant protein allows binding of ATP in site II and the formation of π-π stacking interactions with the adenine ring of ATP, necessary for AZTMP excision [15,17].

Comparison of the AZT resistance mutations selected in HIV-1 and SFVmac shows that in SFVmac no exchange to an aromatic amino acid homologous to T215Y/F occurs. This raises the question as to what the functions of the individual mutations in the AZT resistant SFVmac RT are. We have shown previously that the amino acid exchange I224T is important for viral fitness but not for AZT resistance per se, i.e. it is not required for the AZTMP excision reaction [11,14].

Comparative studies on AZT resistance in ortho- and spumaretroviruses can shed light on the general requirements for AZTMP excision. HIV-1 and SFVmac achieve AZT resistance by AZTMP excision using different, non-homologous resistance pathways. In order to disclose the functions of the individual mutations for AZT resistance and the molecular mechanism in SFVmac PR-RT, we characterized the biochemical properties of various PR-RT variants harboring single, double, triple, and quadruple amino acid substitutions and analyzed the ability of the variants to excise AZTMP. Moreover, we examined ATP binding via NMR spectroscopy.

Here, we determined for the first time the molecular mechanism for AZT resistance in a monomeric RT as well as the dissociation constant for ATP in an AZT resistant retroviral RT.

## Results and discussion

### Protein variants

We have shown previously that the purified SFVmac PR-RTs *mt3* (K211I, S345K, E350K) and *mt4* (K211I, I224T, S345K, E350K) are able to excise AZTMP with similar efficiency from an AZTMP-terminated primer in the presence of ATP [14]. The I224T exchange in *mt4* is probably a polymorphism and does not contribute substantially to AZT-resistance but to viral fitness [11,14]. To reveal the individual contribution of the amino acid exchanges in SFVmac PR-RT for AZT resistance we analyzed various PR-RT enzymes with one to four amino acid substitutions in the polymerase domain. Additionally, all PR-RT enzymes used, carry a mutation in the PR domain leading to the active site residue exchange D24A. This renders the enzymes inactive as a protease. Thus, unintentional PR cleavage can be excluded.

**Figure 1:**
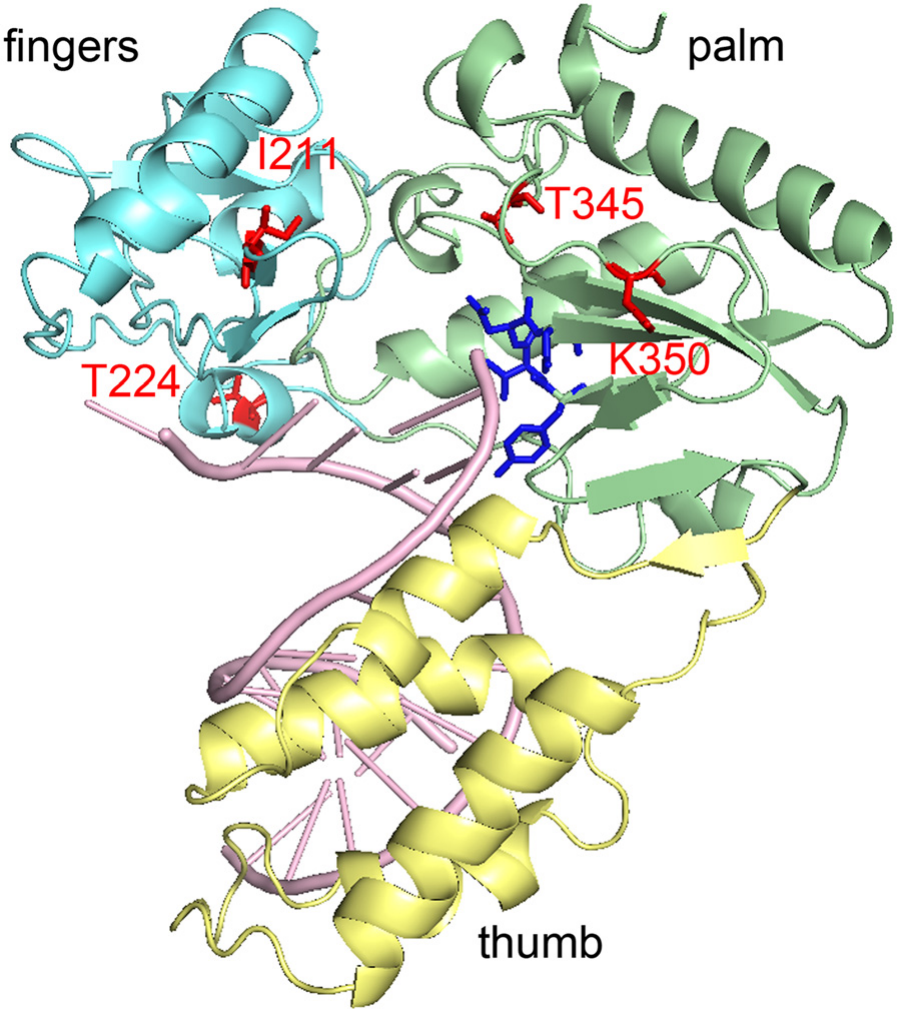
**Localization of the AZT resistance substitutions.** The putative locations of the four substituted amino acid residues in the highly resistant *mt4* (I211, T224, T345, K350) are highlighted in red in a structural model of the SFVmac RT polymerase domain. The polymerase active site motif YVDD is highlighted in blue. The model is based on the structure of XMRV RT (PDB: 4HKQ) with an RNA/DNA hybrid substrate and was generated using the Program SWISS-MODEL [25].

RTs and several other DNA polymerases share a common three-dimensional structure, which is usually compared to the shape of a right hand with the so-called fingers, thumb, and palm subdomains [22-24]. The active site residues are located in the palm, while the thumb and fingers regions wrap around the nucleic acid substrate. Thus, the putative localization of the four exchanged amino acids in AZT resistant SFVmac PR-RT with regard to the polymerase active site could be determined by modeling the structure of the SFVmac polymerase domain with the Program SWISS-MODEL [25] using xenotropic murine leukemia-like related virus (XMRV) RT (pdb: 4HKQ) with a sequence homology of approximately 29 % as a template [22] (Figure 1). Similarly to SFVmac PR-RT this enzyme is also monomeric. The model shows that the polymorphism I224T is located at some distance to the active site residues YVDD, while S345T and E350K are positioned in the palm subdomain next to the active site and thus could be involved in ATP-binding. K211I is part of a loop in the fingers subdomain, homologous to the beta β3-β4 loop in HIV-1 RT. In HIV-1 RT this loop contains the basic amino acids K65 and R72 that interact with the phosphates of an incoming dNTP. These residues have been shown to be important for polymerization activity, drug resistance, and fidelity [26,27].

### Polymerization properties

**Figure 2:**
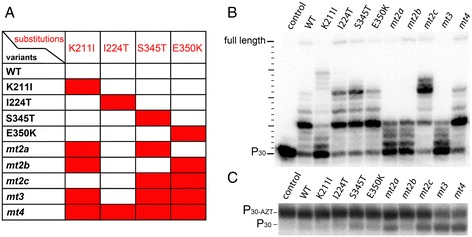
**Polymerization activity and AZT resistance of SFVmac PR-RT variants. (A)**. SFVmac PR-RT variants used in this study harboring one to four of the relevant resistance substitutions are highlighted by red rectangles. **(B)** Polymerization initiation*.* Initiation of polymerization was checked with 20 nM 5′ ^32^P-P_30_/T_50_ substrate, 250 μM dNTPs and 10 nM enzyme at 37°C for 10 min. The PR-RTs used are indicated on top of the gel. WT, wild type; Control, sample without enzyme. **(C)** AZTMP removal in the presence of ATP. 5 nM of an AZTMP-terminated and 5′ ^32^P-labeled substrate P_30-AZTMP_/T_50,_ 0.02 U of pyrophosphatase and 5 mM ATP were preincubated for 5 min. Reactions were started by the addition of 320 nM enzyme as indicated on top and stopped after 20 min. Reaction products were separated on 10% sequencing gels and visualized by phosphoimaging. P_30-AZT,_ AZTMP terminated primer; P_30,_ unblocked primer.

To show that the purified recombinant proteins harboring one to four amino acid exchanges (Figure 2A) were still active as polymerases we determined their specific acitvities on a poly(rA)/oligo(dT) substrate (Table 1). Obviously, the K211I exchange results in severely reduced polymerization activity since the single as well as the double variants harboring K211I (*mt2a, mt2b*) are impaired. In contrast, *mt2c* lacking K211I exhibits high polymerization activity. Remarkably, the specific polymerization activity of E350K is significantly higher than that of the WT.

**Table 1:** **Specific polymerization activities and AZT resistance of SFVmac PR-RTs and corresponding viruses**

**Enzyme**	^1^ **SPAC**	**p-value**	^2^ **AZTMP excision (%)**	^3^ **Viral titer in %**	
				**w/o AZT**	**5 μM AZT**
**WT**	28.6 (±3.1)	-	3.0 (±1.1)	100	<0.1
**K211I**	7.0 (±4.8)	0.005	2.4 (±1.4)	1.2 (±1.2)	<0.1
**I224T**	22.5 (±10.3)	0.410	3.5 (±1.4)	146.5 (±21.4)	0.1
**S345T**	28.8 (±8.3)	0.977	10.4 (±1.2)	40.0 (±16.8)	1.1 (±1.0)
**E350K**	44.7 (±2.1)	0.003	2.8 (±0.7)	31.7 (±8.8)	<0.1
***mt2a***	13.3 (±0.9)	0.009	14.5 (±0.7)	<0.1	<0.1
***mt2b***	8.7 (±2.9)	0.001	7.9 (±0.6)	23.0 (±7.9)	<0.1
***mt2c***	32.6 (±13.8)	0.667	18.7 (±1.2)	20.6 (±5.5)	3.0 (±2.1)
***mt3***	15.5 (±2.6)	0.005	35.4 (±0.6)	8.6 (±2.7)	2.8 (±1.3)
***mt4***	21.7 (±3.9)	0.079	37.9 (±1.3)	113.0 (±19.2)	68.8 (±18.8)

^**1**^SPAC, specific activity in U/10 min*μg_protein_. One Unit (U) catalyzes the incorporation of 1 nmol TTP in a poly(rA)/oligo(dT)_15_ in 10 min at 37°C by 1 μg PR-RT. The values are the means and standard deviations of at least three independent experiments. The p-values were calculated using the unpaired *t*-test in which we compared each variant with the WT. p-values ≤0.05 are considered significant. ^2^Quantification of AZTMP removal depicted in Figure 2C was achieved by phosphoimaging. The amount of unblocked primer after AZTMP excision (P_30_) is equivalent to the quantity of excised AZTMP and is given as a percentage of the total amount of primer in the reaction. ^**3**^Data taken from [11]. The viral titers in the cell free supernatants of the WT in the absence of AZT were set to 100% (≙ viral titer of 7 x 10^4^). Titers of mutant viruses are expressed relative to the WT. Standard errors are given in parenthesis.

Since the specific activities of the variants containing K211I were considerably lower than the WT activities, we performed primer extension assays with a 30/50mer DNA/DNA (P_30_/T_50_) substrate which was 5′ ^32^P endlabeled at the primer. Conditions were chosen that allowed the investigation of the first nucleotide addition steps (Figure 2B) to determine whether the enzymes are impaired in initiating polymerization. Although all proteins were able to extend the primer by a few nucleotides, K211I appeared to be the least active enzyme among the single exchange variants. Accordingly, *mt2a* and *mt2b,* also harboring the K211I substitution, were less efficient in initiating polymerization than *mt2c*. The latter shows an even higher activity than the WT. These data are also reflected in the specific polymerization activities measured with the homopolymeric substrate poly(rA)/oligo(dT) (Table 1). Interestingly, due to the presence of K211I, the activity of *mt3* is reduced as compared to the WT. However, the polymerization behavior of *mt4* is again comparable to the WT owing to the presence of the polymorphism I224T.

### AZT resistance

AZT resistance in SFVmac is based on the removal of the incorporated AZTMP in the presence of ATP [14]. Therefore, to elucidate the function of the individual substitutions for AZT resistance, we tested the AZTMP excision efficiency using a 5’ ^32^P-endlabeled and AZTMP-terminated primer/template substrate (P_30-AZT_/T_50_) and 5 mM ATP (Figure 2C, Table 1). Only one of the singly substituted variants, i.e. S345T, showed significant (>5%) AZTMP excision activity. This indicates that S345T is the key amino acid exchange for AZTMP removal. As shown above, the inability of the other single variants to remove incorporated AZTMP is not due to a lack of polymerization activity (Table 1, Figure 2B). We have shown previously that the single amino acid exchange S345T leads to moderate drug resistance of the virus in the presence of 0.5 – 5 μM AZT. However, in the absence of AZT it resulted in reduced viral titer (40%) as compared to the WT virus [11] (Table 1).

Among the enzyme variants carrying two substitutions, a combination of the key exchange S345T with K211I (*mt2a*) improved the excision efficiency as compared to S345T alone from ca. 10 to 15%, and the combination of S345T with E350K (*mt2c*) almost doubled the excision efficiency (ca. 19%) (Table 1). In contrast, *mt2b*, lacking the S345T substitution, exhibited no significant AZTMP removal activity.

Remarkably, the virus harboring the corresponding substitutions of *mt2a* was not able to replicate, neither in the presence nor in the absence of AZT, although the purified PR-RT variant still showed polymerization activity, albeit reduced (Figure 2B). Virus *mt2c* exhibited moderate AZT resistance, but in the absence of AZT the viral fitness was reduced to ca. 20% as compared to the WT virus [11] (Table 1). These data imply that E350K might be the second AZT resistance mutation, since the combination of the putative first exchange S345T with K211I leads to replication deficient virus (Table 1). The combination of K211I and E350K (*mt2b*) showed little AZTMP excision *in vitro*. This was reflected in cell culture assays with the corresponding virus, which was unable to replicate in the presence of 5 μM AZT [11] (Table 1). Obviously, the K211I exchange on its own and in *mt2a* yields replication deficient virus (Figure 2B, Table 1) [11]. However, in *mt3* the presence of K211I supported AZTMP removal. Compared to *mt2c,* the excision efficiency of *mt3* was about 2-fold higher (Table 1). Moreover, the high excision activity was retained with *mt4*, confirming that I224T improves viral fitness in the fully resistant virus but is not involved in AZT resistance per se. This was also illustrated by the fact that the dramatic decrease of viral titer with the *mt3* virus (8.6% of the WT virus) in the absence of AZT was fully compensated by I224T in the *mt4* virus, bringing the titer back to WT levels (ca. 113%) (Table 1) [11].

Taken together, these data show that the single substitution S345T results in moderate ATZMP excision activity, which can be further improved in combination with K211I or E350K. The major gain in AZTMP excision efficiency is due to the additional exchange K211I. However, this substitution leads to an impaired polymerization reaction, which can be recovered in *mt4* by I224T.

### RNase H activities

**Figure 3:**
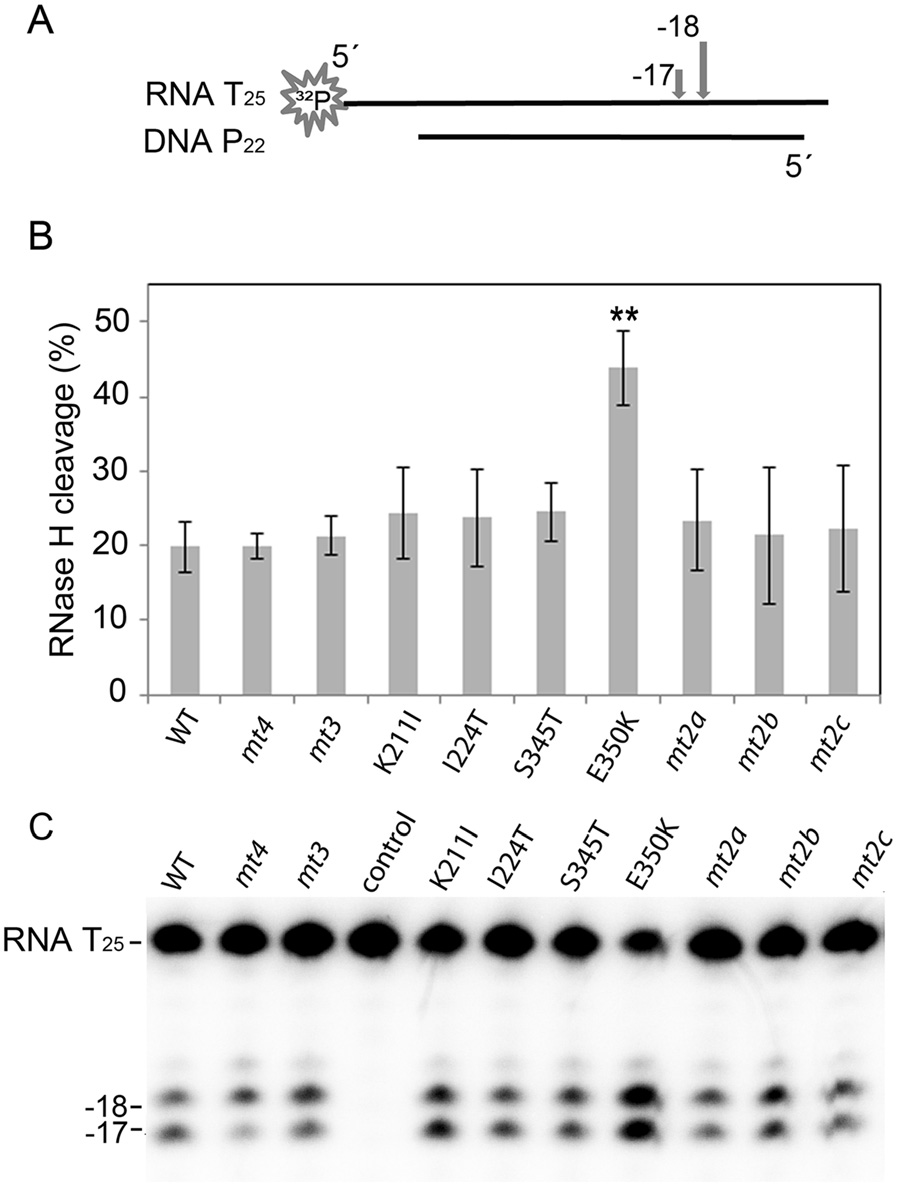
**Qualitative RNase H assay. (A)** Schematic representation of the 5′ ^32^P-RNA_25_/DNA_22_ hybrid. The arrows on top of the RNA indicate the major RNase H cleavage sites at position −17 and −18. The first nucleotide of the RNA hybridized to the 3′-OH nucleotide of the DNA strand is designated −1. **(B)** Quantification of the RNase H cleavage products. The diagram depicts the mean values and standard deviations (black bars) of three independent experiments. For quantification of the cleavage products the total amount of labeled RNA per lane was set to 100%. Only the p-value of E350K ≤ 0.01 (**) represents a statistically significant difference to the WT protein. **(C)** Autoradiogram of a typical RNase H cleavage experiment. RNase H reactions were performed with 240 nM 5′ ^32^P-RNA_25_/DNA_22_ hybrid and 50 nM RT-PR for 2 min at 25°C. RNA T_25_, uncleaved RNA; −17, 18 indicate the cleavage sites; control, reaction mix without enzyme.

In HIV-1 RT certain substitutions in the connection subdomain or RNase H domain leading to a reduced RNase H activity can enhance AZT resistance on a DNA/RNA primer/template. Slowing down the RNase H activity allows the enzyme more time for AZTMP excision to take place [28-30]. To investigate whether the substitutions in SFVmac PR-RT, although not in the connection subdomain or RNase H domain, influence the RNase H activity, we performed qualitative RNase H assays with a 5’ ^32^P-endlabeled RNA_25_/DNA_22_ substrate (Figure 3). However, no significant reduction of the RNase H activities could be detected. Rather, quantification of the cleavage products indicated that E350K exhibits a significantly higher RNase H activity than all the other enzymes. Apart from this effect no significant differences, e.g. in the cleavage pattern, could be detected, implying no influence of the RNase H activity on AZT resistance.

### Kinetic parameters for polymerization and DNA binding

To further assess the effect of the K211I substitution on AZT resistance, we determined the kinetic parameters of the enzymes (Table 2). The reduced polymerization activity of variant K211I might be due to changes in affinities for the nucleic acid substrate and/or the dNTP. Determination of K_M_- and v_max_-values as well as the v_max_/K_M_ ratio for nucleotide incorporation points in this direction. K_M_-values of K211I and variants harboring K211I (*mt2b* and *mt2a*) were about 3-fold (*mt2b*) or 5-fold (*mt2a*) higher than that of the WT enzyme. In contrast, WT v_max_ values are about 4- to 7-fold higher. E350K and *mt2c* reach higher v_max_/K_M_-values than the WT, indicating higher catalytic activities (Table 2). However, this effect was obliterated by the introduction of K211I. The activity of the resulting *mt3* was again similar to the WT.

**Table 2:** **Kinetic parameters of SFVmac PR-RTs for polymerization and DNA binding**

**Enzyme**	^1^ **K**_**M**_ **(μM)**	^1^ **V**_**max**_ **(mM/min)**	**V**_**max**_ **/K**_**M**_ **(1/min)**	^2^ ***K***_**D**_ **P/T (nM)**
**WT**	57 (±7)	2.1 (±0.08)	36.8 (±4.7)	9.6 (±0.7)
**K211I**	160 (±28)	0.3 (±0.02)	1.9 (±0.4)	19.6 (±2.3)
**I224T**	94 (±9)	2.2 (±0.08)	23.4 (±2.4)	8.9 (±1.4)
**S345T**	34 (±6)	1.1 (±0.05)	32.4 (±5.9)	8.7 (±0.9)
**E350K**	32 (±8)	1.8 (±0.10)	56.3 (±14.4)	9.7 (±1.7)
***mt2a***	252 (±39)	0.5 (±0.04)	2.0 (±0.3)	7.8 (±1.2)
***mt2b***	159 (±41)	0.6 (±0.02)	3.8 (±1.0)	15.8 (±2.5)
***mt2c***	9 (±3)	2.5 (±0.10)	277.8 (±92.6)	17.2 (±2.1)
***mt3***	67 (±14)	2.0 (±0.10)	29.9 (±6.2)	15.8 (±2.1)
***mt4***	110 (±11)	1.3 (±0.05)	11.8 (±1.3)	9.5 (±1.6)

^1^K_M_- and v_max_-values were obtained by using the Michaelis-Menten equation to fit a curve to the data. ^2^K_D_-values were obtained as described previously [14] by using an equation for a two state model to fit a curve to the titration data. Standard errors are given in parenthesis.

Fluorescence anisotropy measurements with a fluorescent labeled DNA/DNA P/T resulted in similar K_D_-values for all enzymes (Table 2). These findings confirm that K211I leads to an impairment of polymerization activity due to a decrease in nucleotide binding affinity, but not by altering the P/T binding affinity. Furthermore, substitution E350K and I224T appear to compensate the polymerization deficiency of K211I in the *mt3* variant as well as in the highly resistant *mt4* protein*.*

### Fidelity

**Figure 4:**
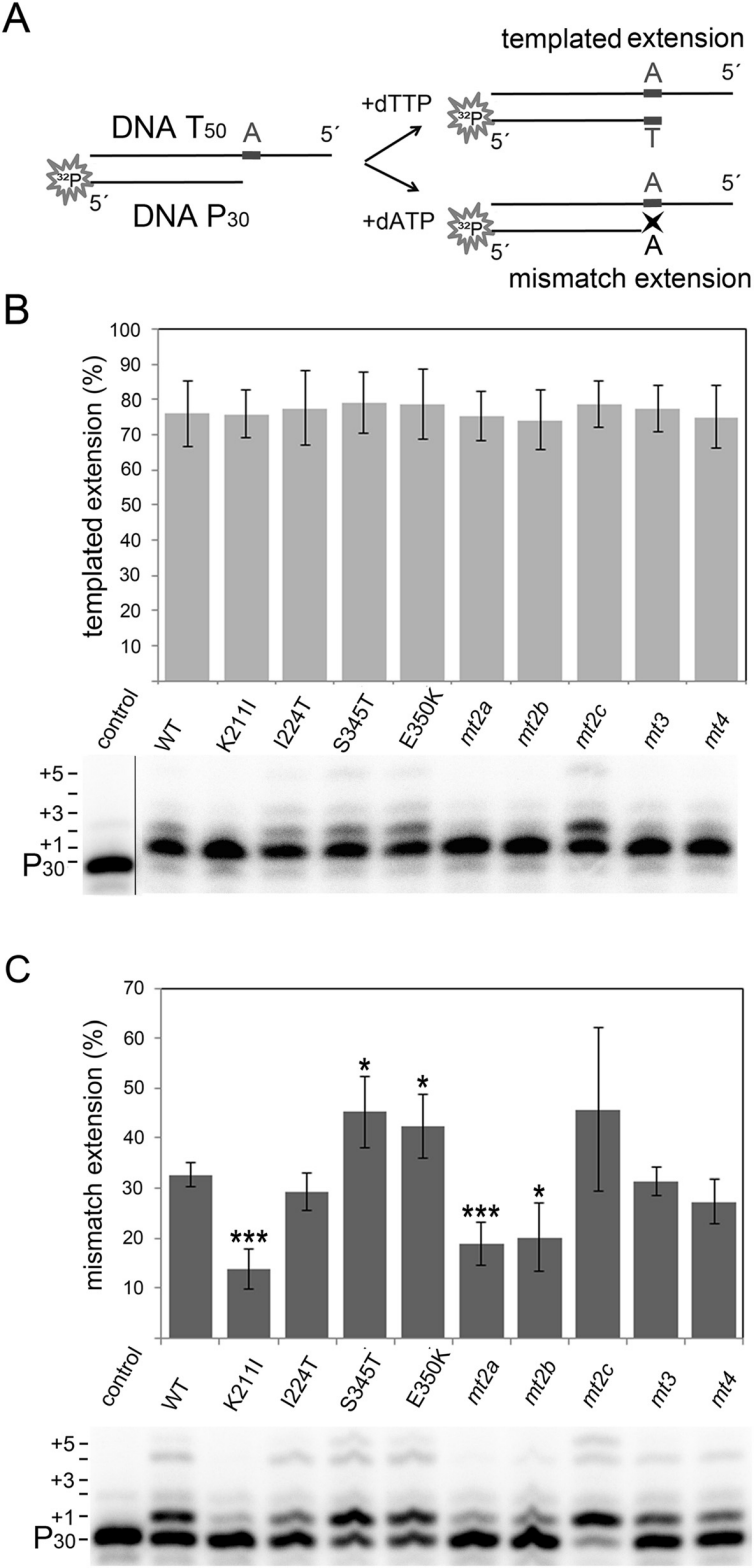
**Fidelity of PR-RTs.** 20 nM of a 5′ ^32^P endlabeled P_30_/T_50_dA DNA/DNA substrate was incubated with 1.25 mM of the correct (dTTP) or incorrect (dATP) nucleotide for polymerization. Reaction products were separated on a 10% sequencing gel. **(A)** Schematic representation of primer extension with the correct nucleotide and primer extension after one nucleotide mismatch. **(B)** Primer extensions in the presence of the next templated nucleotide (dTTP). Control, assay without enzyme. The diagram (top) depicts the mean values and standard deviations (black bars) of three independent experiments. The autoradiogram (bottom) shows the result of a typical extension experiment. The length of the extensions (P_31_ to P_35_) is indicated on the left. Since the bands above P_31_ all comprise the first correct dTTP incorporation they were also included in quantification of the primer extension. For quantification of the extended products the total amount of labeled DNA per lane was set to 100%. **(C)** Mismatch primer extensions in the presence of a non-templated nucleotide (dATP)*.* Evaluation of the results as in **(B)**. P-values ≤ 0.05 represent statistically significant differences to the WT protein (* p-value ≤ 0.05; ** p-value ≤ 0.01;*** p-value ≤ 0.001).

The basic amino acid residues K65 und R72 in the β3-β4 loop region of the HIV-1 RT fingers subdomain not only have an impact on nucleotide binding affinity, but also on fidelity [26,27,31]. The fidelity of a polymerase describes the accuracy of nucleotide incorporation. Therefore, we investigated whether K211I, which is also positioned in the fingers subdomain, alters the SFVmac PR-RT’s fidelity. We used a DNA/DNA P_30_/T_50_dA substrate labeled at the 5’ end of the primer to determine how efficient the primer can be elongated with a complementary (dTTP) or a mismatched dNTP (dATP) (Figure 4A). To overcome the reduced nucleotide binding affinity caused by K211I, a 5-fold higher nucleotide concentration than in the polymerization assay was used for all enzymes.

In the presence of the complementary dTTP all enzymes extended the primer by one nucleotide with similar efficiency (Figure 4B, Additional file 1: Table S1), whereas additional nucleotide incorporations were due to mismatch events. In contrast, when non-templated dATP was used, primer extensions varied considerably (Figure 4C). Quantification of the extended primer after misincorporation disclosed that variant K211I, as well as *mt2a* and *mt2b*, also harboring the K211I exchange, exhibited a significantly higher fidelity than the other enzymes, i.e. reduced incorporation efficiency for the mismatched dATP. *mt2c,* lacking the K211I substitution, showed a behavior similar to the WT enzyme (Figure 4C, Additional file 1: Table S1). In *mt3*, S345T and E350K appear to compensate the effect of K211I, since *mt3*, although containing the K211I exchange exhibited WT fidelity. Due to a decreased binding affinity for all dNTPs in enzymes harboring K211I, dissociation of the incorrect nucleotide and thereby correct base pairing is facilitated. This is similar to the results shown with the substitutions K65R and M184V/I in HIV-1 RT revealing an inverse correlation between fidelity and processivity or activity of the RT [26,27,32-35].

In *mt3* the reduced viral fitness of K211I is improved by S345T and E350K (Table 1), whereas the impaired fidelity caused by S345T and E350K is counteracted by K211I (Figure 4C). However, in order to restore viral fitness completely, the additional exchange I224T, resulting in *mt4,* is required. Thus, some of the mutations in AZT resistant SFVmac RT *mt4* are not absolutely required for AZTMP removal but compensate the negative effects of the substitutions crucial for resistance.

### ATP binding analysis

Interactions of proteins with ligands, e.g. ATP, can be investigated by NMR spectroscopy. An NMR spectrum of a ^15^N labeled protein, which correlates resonance frequencies of amide protons and directly bonded ^15^N labeled nitrogen atoms (2D [^1^H-^15^N] HSQC, *heteronuclear single quantum correlation*) allows the individual detection of peptide amide signals in the protein. Each signal represents a single amino acid of the peptide chain. Chemical shifts are very sensitive to the local structure of the protein. Changes in the chemical environment of amino acids, e.g. due to ATP binding, lead to chemical shift changes in the spectrum which indicate interactions between the protein and ATP.

**Figure 5:**
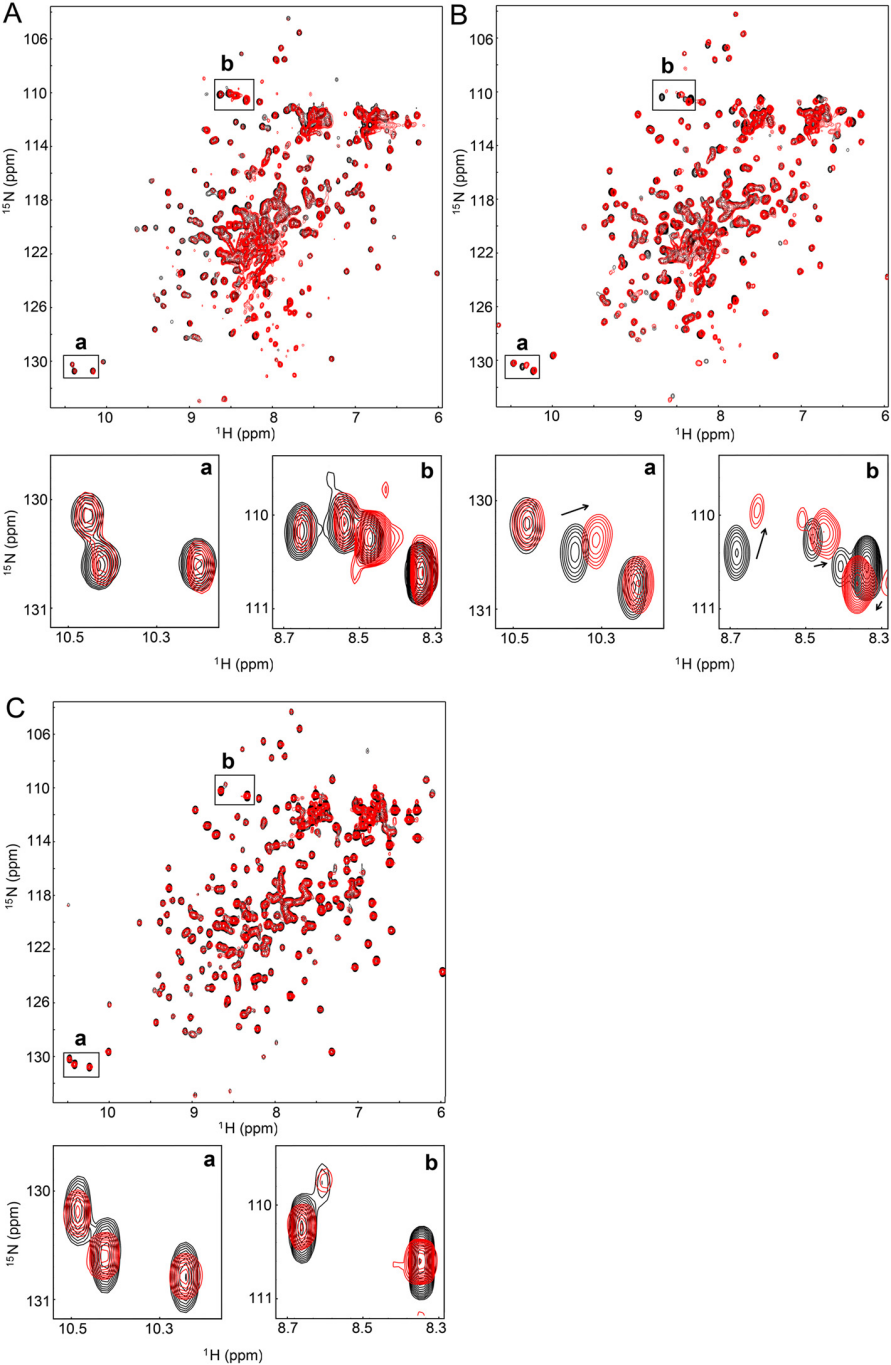
**HSQC spectra of RTshort-WT, RTshort-** ***mt4*** **and RTshort-** ***mt4*** **-T345S** ***.*** Overlays of ^1^H/^15^N-HSQC spectra of RTshort proteins in the absence of ATP (black) and in the presence of 21 mM ATP (red) in binding buffer with 10% D_2_O at a sample temperature of 298 K. **(A)** black: 460 μM RTshort-WT without ATP; red: 363 μM RTshort-WT with a 58 fold ATP excess. **(B)** black: 550 μM RTshort-*mt4* without ATP; red: 434 μM RTshort with a 48 fold ATP excess. **(C)** black: RTshort-*mt4*-T345S without ATP; red: 355 μM RTshort-*mt4*-T345S with a 59 fold ATP excess

Analysis of large proteins by NMR is limited due to extensive signal overlaps and a severe reduction in signal intensity. To obtain an RT small enough and suitable for NMR analysis, we constructed a truncated SFVmac polymerase domain lacking the thumb but comprising the fingers and palm subdomains that contain all four amino acid substitutions for AZT resistance. In previous work, we have already determined the starting point of the RT domain at residue 107 of the PR-RT enzyme. Deletion of the PR at that residue provides a soluble and catalytically active RT [4]. Based on sequence alignments with a catalytic fragment of Moloney murine leukemia virus (MoMLV) RT [36], the corresponding region of SFVmac RT harboring amino acid residues 107–368 (RTshort) was constructed. Determination of its specific activity for polymerization on poly(rA)/oligo(dT) indicated a very low polymerization activity (0.83 U), however, the protein was still able to bind double stranded DNA, albeit with a ca. 100 fold higher K_D_-value (3.6 ± 0.5 μM) than the WT enzyme [3]. The ^1^H/^15^N-HSQC of RTshort-WT recorded in the absence and presence of 21 mM ATP (58 fold excess) revealed no significant chemical shift changes upon ATP addition (Figure 5A), indicating lack of ATP binding, although the concentration is far beyond published physiological values of < 10 mM [37-39].

However, with AZT resistant RTshort-*mt4* several significant chemical shift changes upon ATP addition (48-fold excess) could be observed, demonstrating ATP binding. Since S345T is the only single amino acid exchange exhibiting AZTMP excision activity (Figure 2C), we reversed the S345T exchange in RTshort-*mt4* back to WT (=RTshort-*mt4-*T345S) and recorded ^1^H/^15^N-HSQC spectra in the absence and presence of ATP (Figure 5C). Even at a 59-fold excess of ATP no chemical shift changes were detectable in the spectrum of RTshort-*mt4*-T345S. This result indicates a key role of the S345T substitution in AZT resistance via creating an ATP binding pocket, which is necessary for the excision mechanism.

Assignment of protein backbone resonances by TROSY-based triple resonance NMR analyses using a ^2^H/^15^N/^13^C labeled RTshort-*mt4* sample failed due to insufficient sample stability (precipitation) over the course of the experiments. Using the initially recorded HNCA experiment together with known chemical shift regions, the ^1^H chemical shift signals between 10.0 and 10.5 ppm in the ^1^H/^15^N HSQC spectrum could be identified as tryptophan residues (indol-NH resonances). Typically, the indol-NH-resonances are located in the chemical shift range between 9.5 and 10.5 ppm [40,41]. Comparison of the spectra (Figure 5, boxes a) proves that a Trp residue is involved in ATP binding in RTshort-*mt4,* since a chemical shift change is detectable in the relevant ppm-range (Figure 5B, box a). Obviously, this Trp residue is obscured in RTshort-WT as well as in RTshort-*mt4*-T345S. The chemical shift change of a Trp residue suggests a direct contact of that residue with ATP via π-π interactions, very similar to the interactions between ATP and F/Y215 in HIV-1 RT [15]. In the highly AZT resistant SFVmac PR-RT the substitution S345T might alter the surface exposed residues in its vicinity and thus makes a Trp residue more accessible for hydrophobic interactions with ATP. In contrast to HIV-1 RT, AZT resistance substitutions in SFVmac PR-RT do not result in an aromatic amino acid, but instead make an already existent Trp accessible to fulfill a similar task as the T215Y/F exchange in HIV-1 RT.

### Dissociation constant for ATP binding

**Figure 6:**
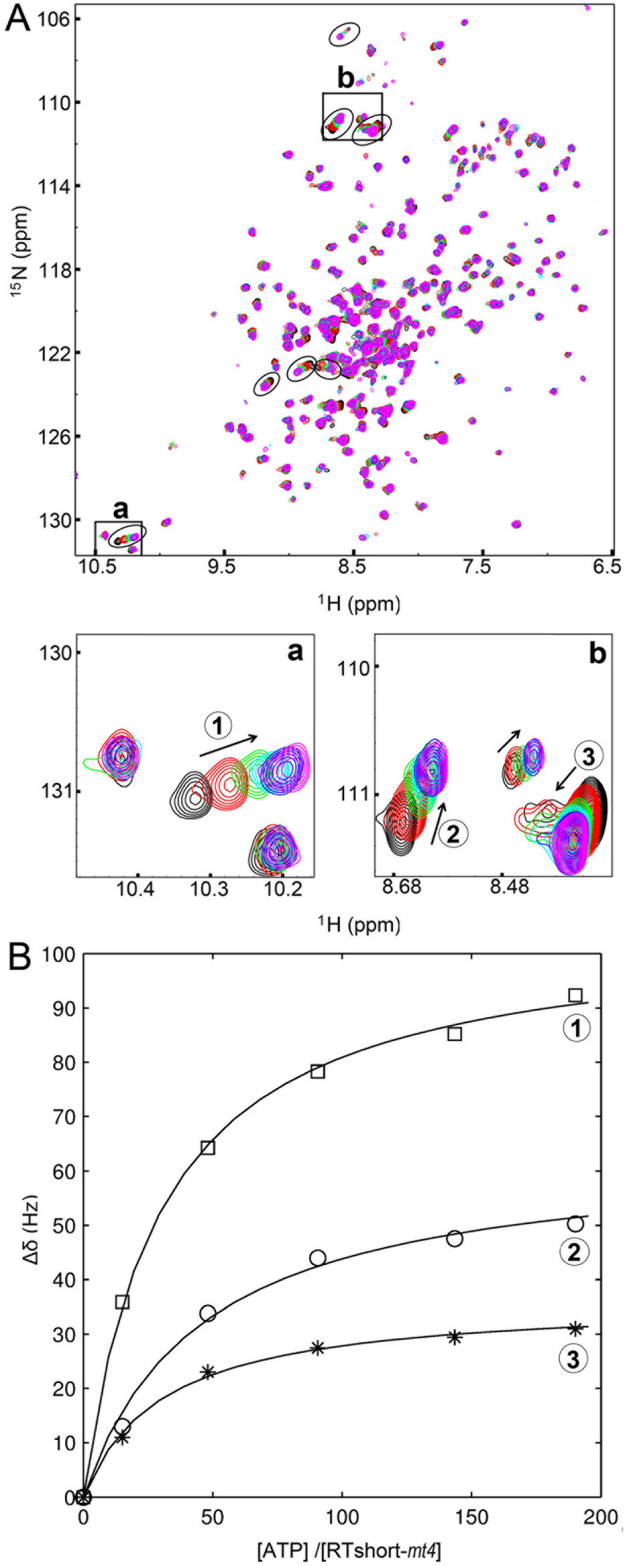
**Titration of RTshort-** ***mt4*** **with ATP** ***.*** **(A)** Overlay of TROSY-^1^H/^15^N-HSQC spectra of 205 μM RTshort-*mt4* recorded at a sample temperature of 288 K during the titration with different ATP/protein ratios. Key: black: 0; red: 15; green: 48; cyan: 92; blue: 142; pink: 195. Blow-ups of the framed areas **(a)** and **(b**) are shown underneath the spectra and represent examples of significant chemical shift changes with RTshort-*mt4* and ATP. **(B)** Determination of the mean K_D_-value for ATP with RTshort-*mt4*. Seven fits for normalized chemical shift changes of seven shifting residues encircled in **(A)** were used to calculate the *mean* K_D_-value with standard deviation. Exemplified, fitted curves for three signal changes depicted in boxes **(a)** and **(b)** are shown as a function of the [ATP]/[RTshort-*mt4*] ratio.

To assess the K_D_-value for ATP binding in RTshort-*mt4* we needed to improve the quality of the spectra. Therefore, RTshort-*mt4* was deuterated in addition to ^15^N labeling to allow the recording of TROSY ^1^H/^15^N-HSQC spectra (Figure 6A). Application of this technique resulted in sharper NMR signals owing to reduced transverse relaxation and rendered faster recording of the spectra possible. Thus, sample stability problems, i.e. precipitation during NMR measurements, could be overcome.

In a series of ^1^H, ^15^N correlation experiments with increasing ATP concentrations, several signals showed the typical gradual change of chemical shifts observed for fast chemical exchange on the NMR time scale. The actual chemical shift in such a spectrum represents the population average of the signal frequencies in the free and bound state, allowing the determination of the dissociation constant in a series of titration experiments. The average K_D_-value for ATP from seven shifting signals in the HSQC spectrum was 7.6 (±2.4) mM. (Figure 5B). This K_D_-value is in the range of published physiological concentrations for ATP in the low millimolar range [37-39].

To analyze how the resistance substitutions alter the ATP binding affinity in the full-length PR-RT enzymes we determined K_M_- and v_max_-values for ATP using varying ATP concentrations in AZTMP excision assays (Table 3). The kinetic parameters were only defined for those enzyme variants capable of excising AZTMP in the presence of ATP (Figure 2C). While K_M_-values for S345T, *mt2a* and *mt2c* were comparable, significantly lower K_M_-values were measured with *mt3* and *mt4*, again indicating that the combination of the three exchanges K211I, S345T and E350K is necessary for high affinity ATP binding and thus for efficient AZTMP removal.

**Table 3:** **Kinetic parameters for AZTMP removal by ATP in SFVmac PR-RTs**

**Enzyme**	**K**_**M**_ **(μM)**	**p-value**	**V**_**max**_ **(nM/min)**	**p-value**
**S345T**	811 (±127)	0.004	110.7 (±6.7)	0.02
***mt2a***	893 (±268)	0.011	112.4 (±14.7)	0.08
***mt2c***	899 (±73)	0.001	108.7 (±11.2)	0.06
***mt3***	142 (±44)	0.226	100.5 (±22.5)	0.14
***mt4***	241 (±101)	-	131.2 (±5.6)	-

The p-values were calculated with the unpaired *t*-test, each variant was compared with the highly resistant *mt4* variant. p-values ≤ 0.05 were considered significant.

## Conclusions

**Figure 7:**
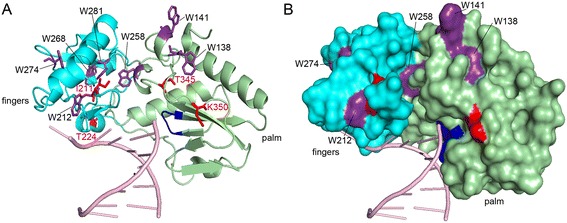
**Localization of tryptophan residues in RTshort** ***mt4*** **.** A structure homology model was generated based on the structure of XMRV RT (PDB: 4HKQ) using the Program SWISS-MODEL. Color coding is analogous to Figure 1. The Trp residues are highlighted in purple. **(A)** Ribbon diagram and **(B)** space filling representation of the fingers and palm subdomains of *mt4*.

We were able to assign different functions to the residues in SFVmac RT responsible for AZT resistance. S345T is probably the first exchange since it is the only single amino acid exchange conferring moderate AZT resistance. Thus, S345T is essential for AZT resistance. It appears to make a Trp residue in the palm subdomain accessible for ATP binding by π-π stacking interactions of the aromatic rings, thus establishing a new ATP binding site in the enzyme variant that does not exist in the WT. Figure 7 shows a model of SFVmac RT *mt4* highlighting the localization of the seven Trp residues that are present in the fingers and palm subdomains. The spacefilling representation (Figure 7B) indicates that most of the Trp residues are located in the inner core of the enzyme and thus do not come into consideration for ATP binding. However, residue W258 is close enough to the active site as well as to the essential residue T345. Furthermore, it is not positioned in the enzyme’s interior and thus might be the Trp residue qualifiying for ATP binding. Our results are in strong contrast to AZT-resistant HIV-1 RT, in which a second ATP binding site is created in the enzyme (site II) ca. 10 Å away from site I, by introducing the aromatic substitution T215F/Y [15-17].

In SFVmac RT the substitution E350K improves the catalytic efficiency of dNTP incorporation, thereby compensating the reduced polymerization activity caused by K211I in *mt3* and *mt4*. K211I increases fidelity by reducing dNTP affinity, thus facilitating dissociation of a non-complementary dNTP.

The K_D_-value for ATP of RTshort-*mt4*, harboring the new ATP binding site, is ca. 7.6 mM. This is in good agreement with published cellular ATP concentrations [37-39] and the ATP concentration (5 mM) we successfully used for *in vitro* AZTMP excision experiments. In HIV-1, the binding sites for ATP and the incoming dNTP overlap [15,42]. Obviously, the affinity for ATP needs to be lower than that for the dNTP since otherwise ATP binding would inhibit polymerization in the absence of AZTTP. Nevertheless, ATP binding can be achieved when AZTMP is incorporated due to pausing of the RT in the so-called pre-translocation complex in which the binding pocket for the dNTP is not accessible [16].

In addition to ATP binding, the excision mechanism further requires formation of a phosphodiester bond between ATP and the incorporated AZTMP, followed by the dissociation of the putative excision product, dinucleoside tetraphosphate (AZT-P_4_-A) [12]. Due to its reduced affinity for nucleotides K211I might facilitate the dissociation of the AZT-P_4_-A, thereby improving the excision reaction in the presence of S345T. Moreover, K211I reduces viral fitness which is at least in part due to the impaired polymerization activity. This, in turn, is compensated by I224T, which improves viral fitness but is not directly involved in the AZT excision process. The amino acid exchange S345T can be achieved by a single nucleotide exchange (TCA > ACA). Therefore, moderately AZT resistant virus is created very easily. This might be the reason why the AZTMP removal pathway is favored over AZTTP discrimination. The functions of the other exchanges are important to obtain highly resistant virus, with improved fidelity and polymerization activity, combined with recovered viral fitness. Thus, although HIV-1 and SFVmac achieve AZT resistance by removal of the incorporated AZTMP, the function of the resistance mutations appear to be different.

## Methods

### Cloning, expression and protein purification

Construction of WT, *mt3*, and *mt4* SFVmac PR-RTs was published previously [14]. The single and double mutants (Figure 2A) were created by site-directed mutagenesis according to the QuickChange protocol from Stratagene (Heidelberg, Germany). Gene expression and protein purification of all SFVmac PR-RT variants was performed using published protocols [14].

SFVmac RTshort-WT, −*mt4 and -mt4*-T345S, coding for amino acid residues 107–368 of the PR-RT, were cloned into the vector pET-GB1a (G. Stier, EMBL, Heidelberg, Germany), expressed as 6His-GB1a fusion proteins, and purified as published previously for prototype foamy virus (PFV) RNase H [43]. In brief, for ^1^H/^15^N HSQC experiments, gene expression in *Escherichia coli* Rosetta DE3 (Novagen/EMD Biosciences; Darmstadt, Deutschland) was induced with 1 mM isopropyl-thiogalactoside (IPTG) at an optical density at 600 nm of 0.7 at 20°C in M9 medium supplemented with trace element solution TS2 [44,45], 1 x MEM vitamin solution (Gibco, Karlsruhe, Germany), 1.5 g/l (^15^NH_4_)_2_SO_4_ (Cambridge Isotope laboratories, Inc., Andover, MA, USA), and 2.5% of ^15^N labeled rich medium (Silantes, Munich, Germany). In order to obtain ^2^H/^15^N- and ^2^H/^15^N/^13^C- labeled RTshort-*mt4* for the TROSY ^1^H/^15^N-HSQC pre-cultures were grown as above or additionally with 4 g/l ^13^C-glucose (Euriso-Top, GIF-SUR-YVETTE, France), using a stepwise increase of D_2_O concentrations from 50% and 75% up to a final concentration of 100%. After cell lysis the proteins were purified by Ni-affinity chromatography. The 6His-GB1a-tag was cleaved off by tobacco etch virus (TEV) protease and the tag was removed using a second Ni-affinity chromatography. The free RTshort variants were collected in the flow through.

### Quantitative polymerization assay

Measurements of RNA-dependent DNA polymerase activity to determine the specific activity (SPAC) were carried out on a poly(rA)/oligo(dT)_15_ substrate (0.2 U/ml) (Roche Diagnostics GmbH, Mannheim, Germany) as discribed previously [14]. Under these conditions 1 unit (U) of enzyme activity enables the incorporation of 1 nmol TMP into poly(rA)/oligo(dT)_15_ after 10 min at 37°C.

To determine K_M_- and v_max_- values, different dTTP concentrations of 25, 50, 75, 125, 250, 600 μM were used in the polymerization reactions and data were fitted to the Michaelis-Menten equation (1) using the fitting program GraFit 5.0.12 (Erithacus Software limited, West Sussex, UK):

1 $$ \mathrm{v}=\frac{{\mathrm{v}}_{\max}\operatorname{}\left[\mathrm{dTTP}\right]}{{\mathrm{K}}_{\mathrm{M}}+\left[\mathrm{dTTP}\right]} $$

### 5′ endlabeling of primers

5′ ^32^P-endlabeling of the P_30_ primer (5′-GCTGTAATGGCGTCCCTGTTCGGGCGCCTC) (biomers.net; Ulm, Germany) as well as the corresponding AZTMP terminated P_30_ primer (P_30-AZTMP_) was done as described previously [3,14].

### Polymerization initiation reactions

Polymerization assays were performed using a 5′ ^32^P endlabeled P_30_/T_50_ substrate (template 5′-GCTGTGGAAAATCTCATGCAGAGGCGCCCGAACAGGGACGCCATTACAGC), 250 μM of each dNTP and PR-RT as described previously [4].

### Qualitative RNase H assay

The RNA template (5′ AACAGAGUGCGACACCUGAUUCCAU) (Metabion GmbH, Planegg-Martinsried, Germany) was ^32^P-labeled at its 5′end and hybridized to a DNA primer (5′ TGGAATCAGGTGTCGCACTCTG) (Metabion GmbH, Planegg-Martinsried, Germany) using a 1.2 fold excess of the DNA [3,14]. RNase H reactions were performed with 240 nM RNA_25_/DNA_22_ hybrid and 50 nM RT-PR for 2 min at 25°C. Reactions were stopped by the addition of an equal volume of urea loading buffer (0.1% bromophenol blue, 0.1% xylene cyanol, 8 M urea, 89 mM Tris/HCl pH 8.3, 89 mM boracic acid) supplemented with 50 mM ethylenediaminetetraacetate (EDTA). Reaction products were separated by denaturing gel electrophoresis (10% polyacrylamide/8 M urea gels). Products were visualized using a phosphoimaging device (Dürr Medical CR 35 Bio; Bietigheim-Bissingen, Germany) and quantified densitometrically with the software AIDA Image Analyzer V.450 (raytest, Staubenhardt, Germany).

### Fluorescence equilibrium titrations

To determine dissociation constants (K_D_) for DNA binding, changes in anisotropy were measured by fluorescence equilibrium titrations with 5 nM of a 24/40-mer DNA/DNA (primer 5′-ATCACCAGGAGAGGGGAAAGCGGA, template 5′-DY647-CTAATTCCGCTTTCCCCTCTCCTGGTGATCCTTTCCATCC) (biomers.net GmbH, Ulm, Germany) and increasing amounts of enzyme at 25°C. K_D_-values were calculated via non-linear curve fitting of the anisotropy data to a two component binding equation as described previously [43].

### AZTMP excision

The P_30_ DNA primer was terminated with AZTTP as described [14]. Subsequently, the resulting P_30-AZTMP_ was purified via gel electrophoresis on a 20% denaturing polyacrylamide/8 M urea gel followed by UV shadowing to identify the DNA. After cutting out of the correct band, the DNA was electro-eluted, dialyzed against H_2_O and lyophilized. 100 pmoles of 5′- [^32^P] endlabeled P_30-AZTMP_ DNA were hybridized to a 1.2-fold molar excess of the T_50_ template DNA as described [4,14]. 5 nM P_30-AZTMP_/T_50_, 0.02 U of pyrophosphatase (Sigma-Aldrich Chemie GmbH, Taufkirchen, Germany) and 5 mM ATP (Jena Bioscience, Jena, Germany) were preincubated for 5 min at 37°C in reaction buffer in a total volume of 10 μl. AZTMP excision reactions were started by the addition of 320 nM enzyme, carried out for 20 min at 37°C and stopped by adding 10 μl of urea loading buffer with 25 mM EDTA. Reaction products were separated by denaturing gel electrophoresis and quantified as described above.

To determine the K_M_- and v_max_-values for ATP excision identical conditions were applied. However, ATP concentrations were varied, using 0.0, 0.15, 0.3, 0.5, 1.0, 2.0 or 3 mM ATP. K_M_- and v_max_-values were obtained using Hanes-Woolf linearization of the Michaelis-Menten equation.

### Polymerase fidelity measurements

A 50mer DNA template (T_50_dA) (5′- GCTGTGGCCGGTCTCTTGTAGAGGCGCCCGAACAGGGACGCCATTACAGC) was hybridized to a 5′ ^32^P-labeled DNA primer (P_30_) (5′-GCTCTAATGGCGTCCCTGTTCGGGCGCCTC) (biomers.net; Ulm, Germany) [4]. 20 nM of the T_50_dA/P_30_ substrate were pre-incubated for 2 min at 37°C with 0.08 U of pyrophosphatase (Sigma Aldrich Chemie GmbH, Taufkirchen, Germany) and 1.25 mM dATP (mismatch) or dTTP (match) in reaction buffer. Reactions were started by the addition of 1.25 μM enzyme and stopped after 12 min with an equal volume of urea loading buffer (see above). The reaction products were separated via denaturing gel electrophoresis (8% polyacrylamide/8 M urea gels) and analyzed as described above. Since the bands above P_31_ all comprise the first correct dTTP incorporation they were also included in quantification of the primer extensions. Mean values and standard deviations of at least three independent experiments were analyzed in unpaired *t*-tests. Significant differences to the WT enzyme are indicated as p-values in Additional file 1: Table S1 and in Figure 4C by asterisks.

### ATP binding experiments

2D [^1^H-^15^N] HSQC experiments were recorded in the absence and presence of ATP (21 mM) (Jena Bioscience, Jena, Germany) with RTshort-WT, RTshort-*mt4* and RTshort-*mt4*-T345S in binding buffer [50 mM Tris/HCl pH 6.7_25°C_, 150 mM NaCl, 6 mM MgCl_2_, 0.5 mM DTT] with 10% (v/v) D_2_O at 298 K. To determine the K_D_-value for ATP of RTshort-*mt4,* transverse relaxation-optimized spectroscopy (TROSY) ^1^H/^15^N-HSQC experiments [46,47] were done with 205 μM of deuterated and ^15^N labeled ^2^H/^15^N RTshort-*mt4* in binding buffer. ^1^H back-exchange of amide protons was achieved by overnight dialysis of the protein against binding buffer at 4°C. Comparison with spectra of ^15^N-labeled protein demonstrated the nearly complete back-exchange of amide protons. A TROSY ^1^H/^15^N–HSQC spectrum of RTshort-*mt4* was recorded without ATP and after the addition of ATP at various molar ratios as indicated in Figure 6. The K_D_-values for ATP of seven different shifting signals were determined by fitting the chemical shift changes of the TROSY ^1^H/^15^N-HSQCs to a two-state model (equation 2). δ_obs_, δ_P_ and δ_PL_ are the chemical shifts for the protein in the ATP-bound and -unbound state, the free RTshort-*mt4*, and the ATP/RTshort-*mt4* complex, respectively. [*P*_*0*_] is the total protein concentration and *r* describes the ration of ATP and protein concentration, [ATP]/[RTshort-*mt4*].

2 $$ {\Delta \delta}_{obs}={\delta}_P+\left({\delta}_{PL}-{\delta}_P\right)\cdot \left[\frac{\left\{{K}_D+\left( 1+r\right)\left[{P}_0\right]\right\}-\sqrt{{\left\{{K}_D+\left(1-r\right)\left[{P}_0\right]\right\}}^2- 4{\left[{P}_0\right]}^2r}}{2\left[{P}_0\right]}\right] $$

The mean K_D-_value with standard deviation was calculated using the independent fits of the seven shifting residues. TROSY based triple resonance experiments [48] were recorded for protein backbone assignment using a ^2^H/^13^C/^15^N labeled sample. All spectra were acquired on Bruker Avance 700 and 800 MHz spectrometers equipped with cryogenically cooled probes. The NMR data was processed applying in house protocols and analyzed with the program NMRView (B. A. Johnson; Merck, Whitehouse Station, NJ, USA).

## Additional file

Additional file 1: Table S1. Fidelity of PR-RTs. Quantification of the primer extension reactions with a templated (match) and non-templated (mismatch) dNTP represented in Figure 3.

## Abbreviations

AZT: Azidothymidine
FV: Foamy virus
GB1a: Immunoglobulin binding domain B1 of streptococcal protein G
HIV-1: Human immunodeficiency virus type 1
IN: Integrase
IPTG: Isopropyl-thiogalactoside
K_D_: Dissociation constant
NRTI: Nucleoside reverse transcriptase inhibitor
NtRTI: Nucleotide reverse transcriptase inhibitor
SFVmac: Simian foamy virus from macaques
MoMLV: Moloney murine leukemia virus, PR, protease
RNase H: Ribonuclease H
P/T: Primer/template
RT: Reverse transcriptase
TEV: Tobacco etch virus
