# Supplementary material for: AZT resistance alters enzymatic properties and creates an ATP-binding site in SFVmac reverse transcriptase
Source: Retrovirology. 2015 Feb 22;12:21. doi: 10.1186/s12977-015-0147-7 (PMC4359774; doi:10.1186/s12977-015-0147-7)
Supplement: Additional file 1: Table S1. — Fidelity of PR-RTs. Quantification of the primer extension reactions with a templated (match) and non-templated (mismatch) dNTP represented in Figure 3. [file 12977_2015_147_MOESM1_ESM.docx]

# Additional file

**Table S1. Fidelity of PR-RTs**

Quantification of the primer extension reactions with a templated (match) and non-templated (mismatch) dNTP represented in Figure 3.

| **enzyme** | **mismatch extension (%)** | **p-value** | **templated extension (%)** | **p-value** |
| --- | --- | --- | --- | --- |
|  |  |  |  |  |
| **WT** | 32.6 (±2.4) | - | 75.9 (±9.2) | - |
| **K211I** | 13.9 (±4.0) | 0.0005 | 75.8 (±6.7) | 0.99 |
| **I224T** | 29.2 (±3.8) | 0.19 | 77.4 (±10.6) | 0.86 |
| **S345T** | 45.2 (±7.1) | 0.03 | 78.8 (±8.7) | 0.71 |
| **E350K** | 42.5 (±6.4) | 0.05 | 78.6 (±9.8) | 0.74 |
| ***mt2a*** | 18.9 (±4.4) | 0.004 | 75.4 (±7.0) | 0.94 |
| ***mt2b*** | 20.1 (±6.8) | 0.03 | 74.2 (±8.4) | 0.82 |
| ***mt2c*** | 45.7 (±16.3) | 0.21 | 78.7 (±6.6) | 0.69 |
| ***mt3*** | 31.3 (±2.8) | 0.51 | 77.3 (±6.6) | 0.83 |
| ***mt4*** | 27.2 (±4.4) | 0.09 | 75.0 (±8.9) | 0.91 |

p-values ≤0.05 differ significantly from the WT.
